# The effectiveness of podcasts in promoting health among young people: a scoping review

**DOI:** 10.1093/heapro/daaf102

**Published:** 2025-07-03

**Authors:** Yixuan Zou, Albie Sharpe, Daniel Demant

**Affiliations:** School of Public Health, Faculty of Health, University of Technology Sydney, 235 - 253 Jones Street, Ultimo, New South Wales 2007, Australia; School of Public Health, Faculty of Health, University of Technology Sydney, 235 - 253 Jones Street, Ultimo, New South Wales 2007, Australia; School of Public Health, Faculty of Health, University of Technology Sydney, 235 - 253 Jones Street, Ultimo, New South Wales 2007, Australia; School of Public Health and Social Work, Faculty of Health, Queensland University of Technology, 88 Musk Avenue, Kelvin Grove, Queensland 4059, Australia

**Keywords:** health-related podcasts, digital health communication, health promotion, public health interventions, young people, scoping review

## Abstract

Health-related podcasts have gained popularity as a digital medium for health promotion, particularly among young people; however, their remains uncertain. This scoping review synthesizes existing evidence on the role of podcasts in shaping young people’s health engagement, focusing on four key dimensions of effectiveness: awareness, knowledge, behaviour change, and health outcomes. The review followed the PRISMA extension for scoping reviews framework, including a systematic search to identify peer-reviewed studies examining podcast interventions targeting individuals aged 14–26 years. Findings indicate that health-related podcasts are an engaging and accessible tool for young audiences, with strong evidence supporting their role in increasing health awareness and knowledge. However, evidence regarding their influence on behaviour change and long-term health outcomes is inconsistent. Podcasts that incorporated interactive elements, expert-driven content, or relatable narratives appeared more effective in sustaining engagement and facilitating knowledge retention. Additionally, variations in podcast length, content style, and delivery format may influence their effectiveness, though these factors remain underexplored. Despite methodological differences across studies, the findings highlight podcasts as a promising avenue for youth-centred health communication. By synthesizing current evidence, this review provides insights into the characteristics that contribute to effective health-related podcasts for young people and underscores their potential as an innovative tool for digital health promotion.

Contribution to Health Promotion

- Health-related podcasts are an accessible and engaging tool for delivering health information to young people.
- This review shows that podcasts can effectively increase health awareness and knowledge among individuals aged 14–26 years.
- The effectiveness of podcasts in changing health behaviours and improving health outcomes remains inconsistent and requires further investigation.
- Podcast characteristics such as content style, expert involvement, and interactivity may influence engagement and knowledge retention among young audiences.
- Findings highlight the potential of podcasts as a digital health promotion tool and provide insights for optimizing their design and implementation.

## INTRODUCTION

### Background

Podcasts have become a popular emerging media format, providing a platform that encompasses a broad spectrum of topics, including news, entertainment, sports, and health (Robins *et al*. 2024). A podcast is a compressed audio file, similar to an on-demand radio show. The term ‘podcasting’ was coined in 2004 by journalist Ben Hammersley, combining the words ‘iPod’ and ‘broadcasting’ (Singer 2019). The podcasting industry has experienced significant growth, with the global number of podcast listeners reaching 546.7 million in 2024, reflecting a year-over-year increase of 7.85% (Backlinko 2025). This growth is expected to continue, with a global projected audience of 651.7 million listeners by the year 2027 (Backlinko 2025).

A key advantage includes convenience, with users able to listen at any time and location, including while undertaking other daily activities, making them valuable tools for disseminating health information during everyday life (Robins *et al*. 2024). Health-related podcasts can convey a broad spectrum of health information, encompassing topics like medical education, nutrition, fitness, and more (Robins *et al*. 2024). Podcasts can provide a medium to simplify complex health topics, making information more relatable and accessible, including through online and downloadable formats (Sutton-Brady *et al*. 2009).

It appears that young people are particularly enthusiastic consumers of podcasts. According to a survey on the overall audio penetration in Australia conducted in 2023, 70% of respondents aged 18–24 claimed they listened to podcasts monthly (Edison Research 2024). In contrast, only 17% of Australians aged 65 and older stated that they listened to podcasts every month (Edison Research 2024). Statistical data indicates that in 2023, the average amount of time spent listening to podcasts per week was 9.0 h among the adult audience (EdisonResearch 2023). The expanding reach of podcasts, combined with the diverse content, highlights the potential of podcasts in promoting health among young people (Tobin and Guadagno 2022).

### Objectives

Prior scoping reviews have highlighted the growing use of podcasts within professional training fields, including medicine and clinical medicine, while also pointing out the potential to be powerful tools for disseminating innovations and evidence (Kelly *et al*. 2022). The recently published scoping review highlights the promising potential of health-related podcasts for health promotion while also indicating that health podcasts are highly engaging and well received (Robins *et al*. 2024). Young people are among the most frequent consumers of digital media, including podcasts, due to their high levels of mobile device use and preference for flexible, on-demand content (Best and Cole 2022). Given the critical developmental period of this age bracket, effective health communication may significantly influence lifelong health behaviours (Inchley *et al*. 2020). Despite this, there is a gap in studies specifically examining the impact of health podcasts on this demographic. While prior reviews have focused on professional training and broader health promotion, research indicates that young people engage with digital media, including podcasts, in unique ways that influence their health-related knowledge, attitudes, and behaviours (Rideout and Fox 2018). To the best of our knowledge, there has not been a comprehensive review of health podcasts specifically targeting a general youth/young audience, indicating a gap in the existing understanding of the effectiveness of podcasts in fostering young people's health-related behaviours.

Consequently, this scoping review aims to assess the effectiveness of podcasts in promoting health among young people. Guided by the Information-Motivation-Behavioural Skills (IMB) model (Fisher and Fisher 1992), effectiveness in this review is defined through four key dimensions: awareness, knowledge, behaviour change, and health outcomes. The IMB model has been widely utilized in behavioural intervention studies across a variety of public health studies, and indicates that health behaviour change is driven by the interplay of information, motivation, and behavioural skills (Chang *et al*. 2014). Therefore, these four dimensions are critical for evaluating the impact of health-related podcasts among young people. To address this, the overarching research question guiding this scoping review is ‘How do health-related podcasts influence awareness, knowledge, behaviour change and health outcomes among young people, and what factors contribute to their effectiveness and engagement?’. The specific objectives of this scoping review are to:

- Assess the effects of health-related podcasts on four key dimensions of effectiveness among young people: awareness, knowledge, behaviour change, and health outcomes.
- Investigate the use, participation patterns, content, length, structure, and various other elements of podcasts pertaining to youth health.
- Identify existing research gaps to inform the future enhancement of youth-focused health podcasts.

The insights obtained may serve to direct future research endeavours, inform both practice and policy to improve the podcast's production and ability to target specific audiences, and facilitate the formulation of more effective interventions that could incorporate health-related podcasts aimed at young people within healthcare and public health.

## METHODS

### Protocol and registration

This systematic scoping review adheres to the Preferred Reporting Items for Systematic Reviews and Meta-Analyses Extension for Scoping Reviews (PRISMA-ScR) (Tricco *et al*. 2018) (see Supplementary File A for PRISMA-ScR Checklist). A research protocol has not been published for this scoping review. The five-stage framework outlined in Arksey and O’Malley (2005) was adhered to:

- identifying the research question,
- identifying relevant studies,
- study selection,
- charting the data,
- collating, summarizing, and reporting the results (Arksey and O'Malley 2005).

The framework represents one of the most frequently utilized and published frameworks in the scoping review literature (Peters *et al*. 2021), and served as a guide for creating inclusion and exclusion criteria, the methodical search approach, the process of screening, the extraction of data, and the presentation of findings.

### Eligibility criteria

To be included in this scoping review, articles needed to satisfy the following six eligibility criteria:

- The research focused on young people, with an average age range of 14–26 years and the majority of participants being in this age group—this ensures that articles that focus on populations that are commonly but not necessarily exclusively young people (e.g. students) would be included. Articles in which the mean age of the research group was outside this age range were excluded.
- Articles concerning podcasts with a health promotion or education intention on any topic.
- Podcasts served either as the primary or were a significant aspect of a larger intervention. Articles that used podcasting for professional education for non-health promotion purposes were excluded.
- The articles are intervention studies based on primary data and have been published in peer-reviewed journals. Purely theoretical articles, literature reviews, opinion pieces, editorials, letters to the editor, and dissertations were excluded.
- Articles published in 2004 and following years. This coincides with the publication of the first (Sullivan 2019). All articles public prior to 2004 were excluded.
- Articles must be published in English. All articles written in languages other than English were excluded from the research.

### Information sources

To identify all potentially relevant literature, we conducted a comprehensive search of several databases, limited to the English language, on 21 August 2024. The databases we searched comprised: Web of Science Core Collection, PubMed, Embase, APA PsycINFO, SocINDEX with Full Text, CINAHL, LGBTQ+ Source, and MEDLINE with Full Text. These databases were selected to ensure a comprehensive and multidisciplinary approach, covering biomedical, psychological, social, and public health perspectives, acknowledging the multidisciplinary nature of research into digital health interventions.

The search retrieved all records from 1 January 2004 to 21 August 2024. The timeframe from 2004 onwards was chosen to capture the emergence and evolution of podcasts as a widely used medium.

### Search strategy

The search strategy was developed collaboratively by the research team, including an experienced epidemiologist and health promotion practitioner (DD) with extensive experience in conducting systematic reviews. The search strategy was constructed around three key themes: (i) Podcasts, (ii) Health Promotion, and (iii) Young People. The search was restricted to the English language on 21 August 2024. The search terms were selected to maximize the sensitivity of the search while maintaining specificity to the review’s objectives. The health promotion component includes a broad array of terms aimed at capturing both general and specific health topics relevant to young people, recognizing that youth health podcasts often cover diverse and overlapping areas of physical, mental and social health, aligning with the multidisciplinary nature of public health promotion. The population component includes a comprehensive set of keywords to capture the wide variety of terms used to describe young people in the literature, ensuring the inclusion of studies focusing on different developmental stages and contexts, from early adolescence to young adulthood.

(‘Podcast’ OR ‘Podcasts’)

AND

(‘Health promotion’ OR ‘Health Education’ OR ‘Sexual Health’ OR ‘Reproductive health’ OR ‘Substance Use’ OR ‘Substance Abuse’ OR Smoking OR Drinking OR Alcohol OR ‘Physical activity’ OR infection OR ‘Infectious Disease’ OR Health OR ‘Mental Health’ OR ‘Psychological Health’ OR ‘Mental Wellbeing’ OR ‘Psychological Wellbeing’ OR ‘Preventive Health’ OR ‘Health Communication’ OR ‘Health Behavio*’ OR ‘Health Literacy’ OR ‘Health Campaign’ OR ‘Community Health’ OR ‘Health Awareness’ OR ‘Sex Education’ OR ‘Drug Education’ OR ‘HIV Prevention’ OR ‘STI Prevention’ OR Nutrition OR Diet OR Obesity OR Exercise OR ‘Stress Management’ OR Anxiety OR Depression OR ‘Suicide Prevention’ OR ‘Body Image’)

AND

(‘Young People’ OR Adolescen* OR Youth OR ‘Young adult*’ OR Teen* OR Student* OR ‘Emerging Adults’ OR ‘School-Aged’).

### Selection of sources

Records from all results were imported into the EndNote 21 reference management tool (Clarivate 2023). Duplicates that were identified by matching the DOI or title, author, year, and journal were excluded. The citations were subsequently collated and reviewed using Covidence systematic review software (Covidence 2020). Two groups of reviewers (D.D., A.S., and Y.Z.) independently screened records based on title and abstract using the standardized eligibility criteria described above. The first reviewer group, D.D. and A.S., screened the articles in the first and second halves of the list in alphabetical order by article title. Y.Z. completed the screening of all the articles as a second independent reviewer. After title/abstract screening, all were reviewed and conflicts resolved by the third reviewer. The full-text screening was conducted independently by the two groups of reviewers and any conflicts were resolved in the same way as in the abstract screening section.

### Data extraction

We used Microsoft 365 Excel for data extraction and storage, and the author (Y.Z.) charted the data table included in the research under the guidance of other authors (D.D. and A.S.). The following information is included in the data table: Author, Article title, Year of publication, DOI/Link to publication, Country where the study was conducted, Population description, Sample size, Recruitment method, Type of study/methodology, Intervention, Data collection and analysis methodology, Podcast description, Summary of study, Outcome—Awareness (Appeal), Outcome—Knowledge (Appeal), Outcome—Behaviour change (Appeal), Outcome—Health outcome, and Comments. These extraction variables were selected because they align with the primary aims of this scoping review. The template was tested on three articles and was agreed on by all authors. Data extraction was subsequently completed for all articles by the first author (Y.Z.) and extracted data was double checked for each article by another author (D.D. and A.S.). Consistent with scoping review methodology all articles that satisfied our inclusion criteria were included, irrespective of their methodological quality (Tricco *et al*. 2016).

### Synthesis of results

The synthesis process involved a detailed review of the full texts by three authors (Y.Z., D.D., and A.S.) to extract and categorize data. To ensure the reliability and accuracy of data extraction, each included study was independently reviewed by at least two authors. Discrepancies in data interpretation were resolved through consensus discussions among all authors. This multi-stage review process helped to validate the consistency and completeness of the extracted data, reducing the risk of misclassification or data omission. Results were organized into two primary categories: descriptive results and reviewer comments.

- Descriptive results
  These results provided an overview of the effectiveness of podcasts in promoting youth health, focusing on four key dimensions:
  - ‘Awareness’: The degree to which podcasts captured youth attention and increased awareness about health topics.
  - *‘*Knowledge’: The extent of knowledge improvement attributed to podcast content.
  - ‘Behaviour change’: Observable changes in health-related behaviours among youth following podcast exposure.
  - ‘Health outcomes’: Tangible improvements in health indicators or self-reported health status.
- ‘Reviewer comments’:

As part of the synthesis process, the first author (Y.Z.) reviewed each included article and provided detailed comments on the study’s findings and methodology. These comments were independently reviewed and revised by the other two authors (D.D. and A.S.) to ensure consistency, accuracy, and clarity. The synthesis of results followed a narrative approach. This approach was selected as it allows for a comprehensive overview of findings across heterogeneous studies, facilitating the identification of key themes, trends and gaps in the current evidence base. This method also emerged as the most appropriate given the limited number of studies identified, enabling a more flexible and context-rich integration of study findings.

## RESULTS

### Selection of sources of evidence

**Figure 1.**
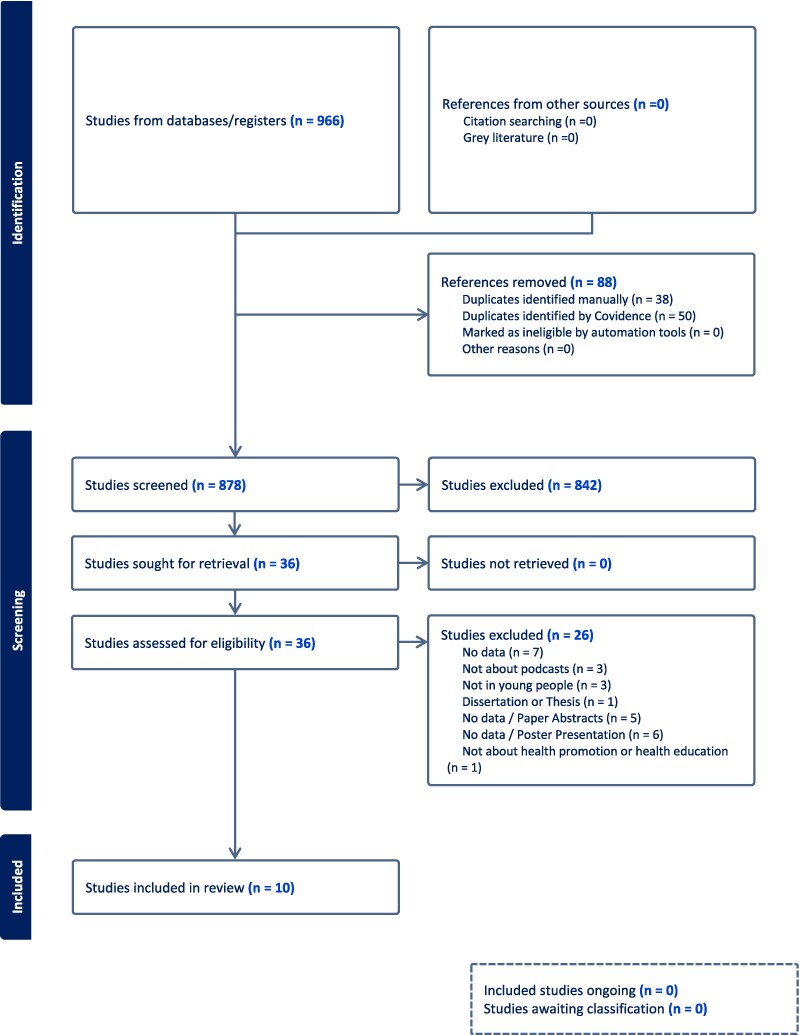
PRISMA flowchart.

A total of 1478 records were extracted from the Web of Science Core Collection, PubMed, Embase, APA PsycINFO, SocINDEX with Full Text, CINAHL, LGBTQ+ Source, and MEDLINE with Full Text. All records were imported into EndNote and deduplicated, leaving 966 records, which were exported to Covidence systematic review software and deduplicated again, finally retaining 878 unique records. We screened the title/abstract for the remaining 878 articles and determined 36 records were retrieved for full text screening. During full text screening, 26 articles were excluded based on various eligibility criteria outlined in Fig. 1, leaving a final sample of 10 articles that matched the inclusion criteria and were included in the current scoping review.

### Characteristics of sources evidence

The extraction data table (Supplementary File B) shows that among the ten articles, four articles described podcasts as an important component of an intervention (Turner-McGrievy *et al*. 2013, Abas *et al*. 2020, Karing 2022, Ajja *et al*. 2024), while the remaining six articles specifically addressed podcasts as the only intervention (Campo *et al*. 2010, Leite *et al*. 2022, Keegan *et al*. 2023, Carrotte *et al*. 2024, Ó Caoilte *et al*. 2024, Taningrum *et al*. 2024).

Among the total articles reviewed, four articles used randomized controlled trials (RCTs) (Turner-McGrievy *et al*. 2013, Karing 2022, Ajja *et al*. 2024, Carrotte *et al*. 2024); two articles used single-arm trials (Abas *et al*. 2020, Leite *et al*. 2022); one article used quantitative method with an experimental research design with a single group with a pre-test post-test (one group with a pre-test post-test design) (Taningrum *et al*. 2024); one article used qualitative research with the focus groups (Campo *et al*. 2010); one article used qualitative research, an open-ended survey with the inductive thematic analysis (Ó Caoilte et al. 2024); one article was a mixed-methods study that primarily involved qualitative, in-depth interviews and a pre-study quantitative questionnaire for interested potential participants. (Keegan *et al*. 2023).

All the articles match the criterion that the study population comprised young people, with an average age between 14 and 26 years old. However, five of the articles did state specific focus on young people but did not provide information on the specific age range (Abas *et al*. 2020, Leite *et al*. 2022, Karing 2022, Ajja *et al*. 2024, Taningrum *et al*. 2024).

### Results of individual sources of evidence

#### Awareness and knowledge

The impact of podcasts on enhancing health awareness and health knowledge among young people was demonstrated with varying degrees of empirical support across the selected studies.

In this study, awareness was defined as the degree to which podcasts captivate young people’s attention and facilitate their initial engagement with health topics, whereas knowledge refers to the improvement in conceptual understanding and retention gained through podcasts and their content. Four articles did not report the impact on awareness and knowledge (Abas *et al*. 2020, Leite *et al*. 2022, Karing 2022, Ajja *et al*. 2024).

Among the reviewed studies, three qualitative investigations revealed consistently positive impacts, each illustrating podcasts engaged young listeners’ awareness and knowledge. Campo *et al*. (2010) demonstrated that the use of humour in podcasts can effectively make sensitive health topics, such as birth control, more accessible and relatable to young listeners. This approach not only enhances their awareness but also encourages them to think critically about their responsibilities and choices in these areas. Keegan *et al*. (2023) similarly identified that podcasts facilitate the formation of personal awareness and knowledge, suggesting that this medium may enable passive listeners to transition into active learners and decision-makers regarding their health. Similarly, Ó Caoilte *et al*. (2024) highlighted that podcasts can introduce new terminology and conceptual frameworks that deepen young listeners’ understanding of mental health. By presenting these topics through diverse narratives and expert discussions, podcasts help listeners develop a more sophisticated understanding and domain-specific vocabulary related to mental health, which can augment their awareness and enable them to discuss and engage with related issues more effectively and confidently.

Moreover, two quantitative studies provided significant statistical evidence supporting the effectiveness of podcasts in increasing awareness and knowledge. Taningrum *et al*. (2024) reported a significant increase in nutrition literacy among high school students, which means the nutritional level with pre- and post-intervention scores rising from 32.81 to 38.15, demonstrating an effective promotion of nutritional knowledge through podcasts (*P* < 0.005). Furthermore, the lower standard deviation in the post-test results of 5.883 showed that post-test data had a more concentrated distribution around the average value and smaller variations compared to pre-test data of 8.832, improving nutritional awareness and knowledge following exposure to the podcast among young people. Carrotte *et al*. (2024) observed that young people's awareness and knowledge of mental illness improved after listening to health promotion podcasts. The Community Attitudes to Mental Illness (CAMI) 27 refers to a 27-item version of the CAMI questionnaire, a widely used psychological tool designed to measure public attitudes and stigma towards individuals with mental illness, comprising 27 questions that assess various aspects of community perception regarding mental health (Sanabria-Mazo *et al*. 2023). There was a statistically significant improvement in the prejudice subscale of the CAMI-27 (*t* = −2.47, *P* = 0.015), which suggests that podcasts could help reduce mental health stigma and improve young people’s awareness and knowledge of mental health.

In contrast, Turner-McGrievy *et al*. (2013) provided a quantitative assessment that showed no statistically significant difference in knowledge acquisition between the podcast and website control groups (*P* = 0.85). This finding suggests that while podcasts may engage listeners more deeply, evidenced by the significantly longer time they spent listening compared to reading content on a website, the method of audio content delivery alone does not guarantee enhanced knowledge. This implies the necessity for careful consideration of methods for podcast content delivery to effectively translate listener engagement into measurable knowledge gains.

These findings collectively illustrate the significant role of podcasts in enhancing health awareness and health knowledge of young people, which still helps establish the groundwork for informed health behaviours and may impact health outcomes among young people.

#### Behaviour change

The effect of podcasts on behaviour change among young people, defined as observable modifications in health-related behaviours following podcast exposure, was inconsistently supported across the reviewed studies. While some studies suggested the potential of podcasts in influencing behaviour, the overall evidence remains mixed, with few studies providing statistical validation.

Among the qualitative findings, Campo *et al*. (2010) suggested that humour-infused podcasts could be a powerful medium for initiating conversations about sexual health, which may subsequently influence young people’s sexual health behaviours. Similarly, Keegan *et al*. (2023) proposed that podcasts might encourage behaviour change and improve health outcomes, yet neither study provided empirical data or statistical validation to support these claims.

Conversely, several studies highlighted limited or no impact on behaviour change. Carrotte *et al*. (2024) indicated that although their podcast intervention affected awareness and knowledge, it did not translate into behavioural intentions. Turner-McGrievy *et al*. (2013) further noted that participants did not exhibit meaningful behavioural improvements, with only a small fraction attempting weight loss and demonstrating interest in the provided materials. Neither study employed statistical testing to measure behavioural outcomes, raising questions about the reliability of these conclusions.

The only study that employed rigorous statistical analysis and directly compared behavioural outcomes between intervention groups found no significant effect of podcasts on behaviour change. Karing (2022) examined the impact of a podcast intervention on young people’s depression management and found no significant improvement within the podcast group [*F*(1, 48) = 0.12, *P* = 0.367], whereas a video-based intervention demonstrated a significant reduction in depressive symptoms [*F*(1, 48) = 7.32, *P* = 0.005]. This suggests that while podcasts may engage young audiences, they may not be as effective as visual-based interventions in prompting behavioural adjustments in mental health contexts.

A notable exception was Abas *et al*. (2020), which reported a statistically significant reduction in stress levels among college students following a podcast-based intervention [*t* = 4.533, *P* < 0.05]. However, it is important to note that this intervention was part of a broader health promotion campaign rather than a standalone podcast study. The integration of multiple intervention components may have contributed to the observed effects, making it difficult to attribute behaviour change solely to podcast exposure. This distinction is critical, as it suggests that podcasts may be more effective when used as part of a multimodal health strategy rather than as isolated interventions.

Taken together, these findings highlight significant methodological limitations in the existing research. Many studies lacked statistical validation or relied on self-reported behavioural intentions rather than objective behavioural measures. Moreover, the variability in intervention designs—ranging from standalone podcasts to those embedded within larger programmes—complicates direct comparisons. While podcasts show promise in engaging young audiences and fostering awareness, their direct impact on sustained behaviour change remains inconclusive.

#### Health outcomes

The impact of podcasts on tangible health outcomes among young people remains inconclusive, with most studies reporting no statistically significant improvements. In this study, health outcomes were defined as measurable enhancements in health indicators or self-reported health status. Among the reviewed literature, four studies did not assess the effects of podcasts on health outcomes (Leite *et al*. 2022, Ajja *et al*. 2024, Ó Caoilte *et al*. 2024, Taningrum *et al*. 2024), while others provided mixed findings, largely indicating minimal or no significant changes.

Several studies qualitatively suggested that podcasts might hold potential for influencing health-related behaviours yet lacked empirical support for their direct impact on health outcomes. Campo *et al*. (2010) proposed that humorous and memorable podcast interventions could facilitate secondary communication, meaning that discussions initiated by podcast content might indirectly contribute to health awareness and knowledge. However, the study acknowledged that no data supported a significant effect on actual health outcomes. Similarly, Turner-McGrievy *et al*. (2013) found that despite podcast exposure, very few participants actively engaged in weight loss efforts, and overall interest in the intervention materials remained low, suggesting that increased exposure to health-related audio content does not necessarily translate into behavioural and health outcomes improvements.

Quantitative studies further reinforced these findings by demonstrating no statistically significant effects on health outcome indicators. Carrotte *et al*. (2024) reported that while the podcast intervention aimed at reducing mental health stigma increased awareness and knowledge, it failed to produce measurable health benefits, as no significant shifts in young people’s behavioural and health outcomes were detected. Karing (2022) similarly found no significant reduction in depressive symptoms among podcast listeners [*F*(1, 41) = 0.03, *P* = 0.437], whereas the control group experienced a significant improvement in life satisfaction [*F*(1, 41) = 15.99, *P* < 0.001], further emphasizing the limited capacity of podcasts to induce direct psychological health benefits.

The commonality across these studies lies in their indication that while podcasts may engage young audiences and foster awareness and knowledge, they often fall short in producing measurable improvements in health outcomes. One possible explanation is that awareness and knowledge acquisition alone do not automatically translate into behavioural changes, especially in the absence of long-term health intervention strategies. Furthermore, differences in podcast intervention design may have contributed to the observed variability; some studies assessed standalone podcast interventions, while others incorporated them into broader health initiatives.

Taken together, these findings suggest a fundamental disconnect between health promotion with podcasts and actual health improvements. While podcasts are effective in disseminating health information and fostering awareness, their ability to drive measurable health outcomes appears to be limited. A key factor may be the passive nature of podcast consumption, where listeners absorb information without necessarily acting on it. Unlike interventions that incorporate interactive elements, structured goal-setting, or professional guidance, standalone podcasts may lack the reinforcement mechanisms required to sustain long-term health improvements. Furthermore, the variation in study designs, ranging from standalone podcasts to those embedded in broader interventions, complicates direct comparisons and may account for the inconsistency in reported outcomes. These findings underscore that while podcasts have value as educational tools, their role as interventions for improving health outcomes remains largely unsupported by current evidence. It also could be that different topic areas engage different audiences with different health concerns. Those listening regarding mental health concerns may be less likely to act (i) because they do not experience mental health issues or (ii) because their mental health issues demotivate them from acting. It may be that audiences interested in sexual health education content may be more engaged to act.

#### Length of podcast

The length of a podcast is a potential factor influencing its effectiveness in health promotion, although it has received relatively limited attention in the existing literature (Prakash *et al*. 2017). Podcasts can vary in duration and are often categorized as short (1–5 min), moderate (6–15 min), and long (>15 min) (Carvalho *et al*. 2009). Matava *et al*. (2013) found that while some listeners preferred a 15- to 30-min format, the majority favoured shorter lengths between 5 and 15 min, a preference echoed by On Tam (2012) in a survey of learners outside graduate medical education.

Among the studies reviewed, three articles explicitly described the duration of the health-related podcasts under investigation. The PsychTalks podcast had an average runtime of approximately 20 min (Carrotte *et al*. 2024), while The Sex Wrap podcast episodes ranged from 20 to 45 min (Keegan *et al*. 2023). In contrast, The Weight Loss podcast, a first-person narration format, was considerably shorter, with an average duration of 4 min and 30 s (Turner-McGrievy *et al*. 2013). However, despite these variations in length, our analysis did not identify any discernible impact of podcast duration on health promotion outcomes.

This evidence suggests that other factors, such as content relevance, delivery style, and audience engagement strategies, may play a more significant role than duration alone. The preference for shorter podcasts in previous listener surveys (On Tam 2012, Matava *et al*. 2013) contrasts with the actual length of health-related podcasts in this review, which were often longer. The diversity in podcast length across studies suggests that duration is likely influenced by the nature of the health topic being discussed, with some topics requiring more extensive discussion than others. While podcast length remains a relevant consideration in health communication, its role as a determining factor in effectiveness remains unclear based on current evidence.

#### Professionalism of podcast content

The level of expertise in health-related podcasts for young people plays a critical role in ensuring the accuracy and reliability of the information delivered. This is particularly important when addressing sensitive health topics, as misinformation or a lack of expertise in content creation could lead to misunderstandings and potentially harmful behaviours (Caoilte *et al*. 2023). Given that young audiences may not always have the ability to critically assess the credibility of health information, the professionalism of podcast content could, in theory, influence its effectiveness in health promotion.

Two studies explicitly examined the professionalism of health-related podcasts targeted at young people, highlighting the role of expert involvement in content production (Keegan *et al*. 2023, Carrotte *et al*. 2024). The PsychTalks podcast, produced by the School of Psychological Sciences at the University of Melbourne, was characterized by its professional recording and editing, with structured segments accompanied by narration and music (Carrotte *et al*. 2024). Similarly, The Sex Wrap, an evidence-based sexual health podcast aimed at individuals aged 14–25, was developed and hosted by two sexologists with PhDs in sexology, education, and sexual health-related subjects (Keegan *et al*. 2023). These podcasts reflect a high degree of professionalism, as their content was curated and presented by qualified experts, distinguishing them from non-expert or user-generated health-related podcasts.

Despite these observations, our analysis found no discernible association between podcast professionalism and its impact on awareness, knowledge acquisition, behaviour change, or health outcomes among young listeners. Although both PsychTalks and The Sex Wrap were professionally produced and hosted by qualified experts, there was no statistical evidence to suggest that their level of professionalism significantly influenced health promotion outcomes compared to podcasts without expert involvement. This suggests that the presence of expert content creators and high production quality alone may not be sufficient to drive measurable health improvements among young audiences.

#### Participation patterns and styles in podcast

Podcast participation patterns and content styles are important factors influencing audience engagement, with prior research suggesting that dialogue-based formats, conversational tones, personal anecdotes, and humour enhance the educational experience by making content more engaging and enjoyable (Riddell *et al*. 2020). This aligns with findings from two studies in the current review, both of which highlighted the role of interactive and entertaining podcast styles in shaping young listener engagement.

The MTSS podcast, an online video podcast focused on sexual health, aimed to present information in a clear and entertaining way while promoting healthy sexual behaviour (Campo *et al*. 2010). Episodes were released monthly and covered topics such as birth control, pornography, and orgasm, utilizing humour as a primary strategy to make these discussions feel more comfortable and approachable. The incorporation of interactive features further aimed to increase engagement by fostering a sense of involvement among listeners. Similarly, The Sex Wrap podcast adopted a conversational and relatable discussion style, integrating elements of popular culture, social movements, politics, and current events into sexual health education (Keegan *et al*. 2023). A distinguishing feature of The Sex Wrap was its direct engagement with listeners through interactive mechanisms, including phone, email, and social media submissions, allowing users to contribute questions that shaped the podcast’s content. This participatory approach ensured that the podcast remained relevant and responsive to audience interests.

Despite the emphasis on participation and interactive styles in these podcasts, our review found no significant association between podcast participation patterns and health promotion outcomes. While dialogue-based and engaging formats are often cited as key factors in effective educational delivery, the current evidence does not indicate that such formats lead to measurable improvements in behaviour change and health outcomes. One possible explanation for this disconnect is that while interactive and humorous content may increase engagement and enjoyment, engagement alone does not necessarily translate into long-term behaviour change. Additionally, differences in podcast content quality, depth of discussion, and the way health messages are framed may play a more influential role than podcast participation style alone.

## DISCUSSION

### Overview of key findings

This scoping review aimed to provide a comprehensive synthesis of the existing scientific evidence on the effectiveness of podcasts in promoting health among young people. The studies reviewed encompassed a wide range of health topics, including sexual and reproductive health, mental health, nutrition, and weight management. While preliminary evidence suggests that podcasts can effectively enhance health awareness and knowledge, their impact on behavioural modification and tangible health outcomes remains inconclusive. This scoping review identified only 10 studies that met the inclusion criteria. This small sample size reflects the emerging nature of this research field, where the body of available studies is still developing. As a result, the findings presented here should be interpreted with caution, as they may not capture the full diversity of podcast interventions or the potential variations in their effectiveness across different contexts.

Approximately half of the included studies indicated that podcasts contributed to greater awareness and knowledge of health topics. This finding aligns with the nature of podcasts as an accessible and engaging medium for health information dissemination. However, evidence supporting behavioural change and measurable health outcomes was considerably weaker. Only one study demonstrated a statistically significant positive impact on behaviour change and health outcomes in the context of a mental health intervention.

Nevertheless, the evidence regarding behavioural modification and long-term health improvements remains inconsistent. Several studies assessing the influence of podcasts on weight loss, adherence to contraception use, and mental health self-care did not report statistically significant changes. While podcasts may facilitate knowledge acquisition, they may lack the necessary reinforcement mechanisms to drive sustained behavioural change. Podcasts may have a significant potential as health promotion tools. However, overreliance on digital platforms may contribute to technology fatigue, reduced face-to-face interactions and an increase in passive learning. Additionally, the lack of professional oversight in some podcasts may raise concerns about misinformation, which may be particularly problematic for young people who may struggle to differentiate between evidence-based content and personal opinions.

### Reviewer comments: quality of included studies

This review included all studies that met the predefined inclusion criteria, regardless of their methodological rigour. However, substantial variations in methodological quality were observed across the included studies, with inconsistencies in study design, deviations in research protocols, and incomplete statistical analyses. Cross-study comparisons were complicated by these variations in study design including differences in intervention formats and measurement tools. Several studies lacked statistical testing, reducing the reliability and reproducibility of their findings, while others suffered from small sample sizes, limiting the generalizability of their results. It was also worth mentioning that some of the studies did not specifically evaluate the podcast components of a more comprehensive health-related programme.

Moreover, several studies relied heavily on self-reported data, introducing a higher risk of response bias and reducing the objectivity of outcome measures. Additionally, the use of convenience sampling limits the generalizability of findings, since participants who regularly engage with health podcasts may differ systematically from the broader population of young people. The lack of objective health indicators or physiological assessments further constrained the ability to draw definitive conclusions about the actual impact of podcast interventions. Additionally, in qualitative studies, the absence of reflexive statements weakened the trustworthiness of data interpretation. Inconsistencies in outcome measures across studies further complicate the assessment of podcasts’ effectiveness in enhancing health awareness, facilitating behaviour change, and yielding measurable health improvements. Finally, all identified studies lacked longitudinal follow-up. This lack of longer follow-ups in the current body of evidence limits our ability to assess the sustained impact of podcasts given that health behaviour change is commonly a gradual and non-linear process that often requires repeated reinforcement and ongoing support to move individuals from precontemplation to maintenance. Without long-term follow-up, it is impossible to understand the real effectiveness of podcasts in supporting the adoption and maintenance of healthier behaviours.

### Discussing the strengths and limitations: identify existing research gaps and deficiencies

Our study is the first research to review the evidence regarding health-related podcasts for the population of young people. Strengths of our study include its highly comprehensive approach, encompassing health-related podcasts’ characteristics and effectiveness. We adhered to a highly robust scoping methodology, which followed PRISMA-ScR guidelines and the Arksey and O'Malley framework (Arksey and O'Malley 2005, Tricco *et al*. 2018).

It is also necessary to acknowledge the limitations of the study. We exclusively concentrate on publications in the English language; it is possible that relevant studies that have been published in other languages have been missed. Similarly, we did not specifically assess the influence of cultural, social or geographic contexts on podcast use and impact. Given the small sample size and limited geographic diversity of the included studies, it is inappropriate to draw firm conclusions about how these factors may shape the effectiveness of podcasts as health promotion tools. Further study limitations relate to the current state of the scientific body of evidence regarding health podcasts. At present, the efficacy data predominantly consists of single-group studies and small RCTs, which introduces potential biases and limits the generalizability of the findings. Additionally, the high level of heterogeneity in terms of study designs, podcast topics and formats, and outcomes across the included studies limits the ability to draw firm conclusions. Finally, it should be noted that for some of the studies, testing the effectiveness of the podcasts in changing attitudes and improving health behaviours, was not the main concern. The further limitation is the unknown quality of related articles, as the articles included in this scoping review vary in methodological quality, posing a potential risk of weakening the research credibility if the information provided is inaccurate or misleading. It is important to recognize that evaluating the effectiveness of podcasts in changing attitudes and improving health behaviours was not the primary objective of some studies. Many studies focused on knowledge acquisition or audience engagement, rather than behavioural modifications or long-term health outcomes, which may partially explain the inconsistent findings across studies. However, by including only published data, we ensure that our review is based on peer-reviewed studies, which enhances the reliability of our findings.

### Future research directions: inform the future enhancement and refinement of podcasts focused on the health of the youth population

Findings from this review highlighted that health podcasts achieve strong engagement among young people, are widely accepted, and may lead to measurable improvements in health awareness and knowledge. However, the current evidence base is still in its formative staged with significant gaps in knowledge that limit the identification of specific needs for future research. The limited number of studies, methodological weaknesses, and heterogeneity in study designs make it challenging to draw definitive conclusions about the most effective approaches for podcast-based health interventions. This early stage of research requires a cautious approach to developing guidance, as premature conclusions risk directing future efforts towards potentially less effective strategies. The following broad areas are identified as valuable directions for future research and practice, based on the current evidence and findings from this review:

- Experimental studies investigating the impact of key podcast characteristics, such as length, content professionalism, participation patterns, and presentation styles, are needed to determine their effects on young people’s health behaviour change and health outcomes.
- Comparative research is needed to assess the effectiveness of podcasts relative to other digital and traditional health communication formats, such as video content, social media campaigns, and written educational materials, to better understand their comparative advantages and limitations in promoting health among young people.
- Large-scale RCTs with long-term follow-up are necessary to provide more rigorous and generalizable evidence regarding both the short- and long-term health impacts of health-related podcasts on young populations.
- Further research should explore the optimal design and structure of podcasts tailored to different health topics and target populations, including evaluations of content quality, delivery format, and listener engagement strategies.

## CONCLUSION

In conclusion, this scoping review assesses the effectiveness of health-related podcasts among young people for health promotion. While the current evidence base is limited, findings suggest that health podcasts can be highly engaging and are generally well received by young audiences. However, their effectiveness in promoting health remains variable, particularly in influencing health behaviour change and long-term health outcomes. While some studies indicate a positive role in enhancing health awareness and knowledge, inconsistencies in study designs and methodologies highlight the need for further investigation. There is a clear need for future research to fully leverage the unique benefits of podcasts for public health. Large-scale, methodologically robust research is needed to substantiate the long-term impacts of health-related podcasts on different populations and maximize their potential as a tool in health promotion, behaviour change, and health outcome interventions.

## Supplementary Material

daaf102_Supplementary_Data

## Data Availability

The data underlying this article are available in the article and in its online Supplementary material.
